# Milk Inspection in Cities

**Published:** 1890-01

**Authors:** W. M. Yandell

**Affiliations:** Health Officer, Health Physician, El Paso, Texas


DANIEL'S

### TEXAS MEDICAL JOURNAL.

A Representative Organ of the Medical Profession, and an Exponent of Rational Medicine; devoted to the Organization, Advancement and Elevation of the Profession in Texas.

PUBLISHED MONTHLY.—SUBSCRIPTION $2.00 A YEAR.

Vol. V. AUSTIN, JANUARY, 1890. No. 7.

### Original Articles.

CONTRIBUTED EXCLUSIVELY TO THIS JOURNAL.

*The Articles in this Department are accepted and published with the understanding that we are not responsible for, nor do we indorse the views and opinions of the writers by so doing.*

For Daniel's Texas Medical Journal.

### MILK INSPECTION.

BY W. M. YANDELL, M. D., HEALTH PHYSICIAN, EL PASO, TEXAS.

SYSTEMATIC inspection of milk is not practiced in Texas, outside of El Paso, so far as I can learn, and as I have put a stop to the sale of adulterated milk here, an account of the simple methods employed, may not only prove interesting to your readers, but assist authorities elsewhere in accomplishing like results.

In the first place, the officer who proposes to inspect milk, must not only prepare himself to make inspections, but to pass a rigid cross-examination as an expert before the courts. By writing to Edward W. Martin, the accomplished and obliging chemist to the Board of Health of the city of New York, he can procure copies of the celebrated Schrumpf case, of Martin's reports for 1884 and 1885, on "Milk and its Adulterations," and of the Sanitary Code of New York city. From these he can learn all that is necessary to be learned from books. Through Mr. Martin he can also procure a tested lactometer, with sworn certificate as to accuracy, the necessary cream gauges, and a tested, sensitive thermometer, if he cannot conveniently procure the latter from the signal service officials.

Having procured these necessary articles, he should go to every dairy furnishing milk to his city, see the cows milked and take a sample of the milk, mixed together from all the cows in the herd, or the mixed milk from at least ten, selected at random, for bear in mind, it is AVERAGE milk, that is, the milk taken from a number of cows and mixed together, that we are particularly interested in. He should also test the milk from single cows, kept by persons for their own use, for every bit of this experience will be valuable to him. In testing he will note the odor, the taste, the consistence of the milk as it runs off the bulb of the lactometer, and its color. He will then set it aside in a cream gauge in a refrigerator, or where it will be kept at a temperature of about 60° Fahrenheit, and after some twenty-four hours skim it thoroughly and take the specific gravity of the skimmed milk, also noting the cream percentage. The cream gauge is an unreliable guide as to richness of milk, but it is a "check" experiment at least. He will also frequently add one part water to one of milk, to two, three, four, five or ten of milk, and take the specific gravity of the mixture, set it aside in the cream guage, allow the cream to rise, skim it and test the specific gravity of the skimmed milk. Having already learned the specific gravity of pure skimmed milk, he will learn from the last experiment that water added to milk stays with the skimmed milk and does not rise with the cream, at least such is my conclusion after a number of experiments. This is a test of my own, and I make it for the following reasons: In his testimony for the defense in the Schrumpf case, Prof. Doremus says: "It is this cream that damns the lactometer." "If the illustrious men of the Board of Health can eliminate the cream, then the lactometer is a good instrument, but with the cream it is impossible with the lactometer to tell whether milk is low from cream or low from water." While the cream gauge is not an exact guide to the richness of milk, as cream does not always rise alike, yet so large a part rises as to practically "eliminate the cream." Should the milk have been low from richness of cream, it should certainly be high after the cream has been removed, but if it was low from water the skimmed milk will be comparatively low. I am inclined to believe that added water causes the cream to separate more thoroughly from the milk.

By these experiments he will have arrived at the highest, the lowest and the average lactometric standing of average milk in his vicinity; from Martin's reports the same facts as to New York, the result of some 20,000 tests, also the lowest and highest of single cows.

Should his city charter give the city council authority, as our city charter does, to pass an ordinance modeled on provisions of the New York City Sanitary Code, it should be passed as prosecutions under the State law, before the county court waste much time. I have, however, secured convictions in such cases after much delay.

He is now ready to sally forth against the milk-men, for, as Martin says in New York, I can say in El Paso, "Whatever may have been used, we find at the present day that the addition of water and the removal of cream, or both, constitute ninety-nine per cent. of the frauds. Carbonate and bi-carbonate of soda, borax and nitrate of soda are sometimes used as preservatives; annatto, butter color, and burned sugar to color the milk, and salt and sugar to impart a taste and to increase the specific gravity, are probably the only adulterants used at present." The popular idea that the milk-men have some secret formula by which they can adulterate milk so as to render detection difficult, is erroneous. I have yet to find one that understands so simple a thing as floating a lactometer properly, or that knows that the specific gravity of milk varies with temperature. The honest ones do not care to.

At first, frequent inspections are necessary, in case the milk-men are adulterating the milk, and this he may be sure most of them, if not all, are doing where inspection is not practiced.

Those who would be honest are forced to quit the business or adulterate their milk to compete with the dishonest.

Should he suspect milk, he will take a sample, test it carefully, and GO THE SAME DAY to the dairy from which it came, see the cows milked, take a sample of the average milk, test and compare it with the suspected milk. When I hit upon this plan, which I have not seen suggested elsewhere, I had no longer any doubts as to success, as average milk varies but a few degrees from one milking to the next.

The 100 point on the lactometer, equal to a specific gravity of 1.029, is taken to represent pure milk, because average milk sometimes falls so low, but in New York the average lactometric standing of milk, the result of testing some twenty thousand specimens, is 110, while the average here from 175 cows is over 115. The lowest single cow's milk here was 106; lowest dairy milk 109.

The first cases that I had were in the county court, against Fink and Doane, who had made probably $40,000 in their business in a few years, and were then running the largest dairy here. Following is the record on which I prosecuted them, this before I hit upon the plan of going to the dairy on the same day that I test suspected milk:

At dairy, October 26, '88, milk from 9 cows, 114; October 29, '88, milk from 16 cows, 118. On the street, October 29, '88, it was 67; October 30, 75; November 19, 75. They pleaded guilty in one case each, and I had the others dismissed. I promised the milk-men on the first day that I inspected that if they would reform I would not prosecute for what I found that day. One whose milk stood at 92 on the street on October 29, 1888, and at 116 at his dairy on October 25, 1888, acknowledged that the 92 milk was watered, but was left off, as subsequent tests showed that he had reformed. Another had milk on the streets on October 30, '88, that stood at 98, while at his dairy October 15 and 16, '88, it stood at 116. He told me the next day after I took the 98 milk that water had been left in the can by mistake. I inspected no more milk until the ordinance went into effect, early in October, 1889. On October 3, 1889, Fink and Doane's milk on the street was 72; same day at the dairy, from two cows (the others were milked before I got there) it was 114. On October 5, on the street, it was 80. I informed the driver on the 5th that I should file complaints against them for selling adulterated milk on the 3d and 5th. On the 6th their milk on the streets was 115, and it kept about that figure daily until about the 10th, when they closed out to an honest firm, and quit the business. When their cases were called for trial, October 12, they pleaded guilty, and were fined in one case each, and the others were dismissed, as they were no longer in the business.

I prosecuted another man whose milk on the streets stood at 86 on October 14, 1889, and on the same day, at his dairy, at 110. He paid a fine, and quit the business in a few days.

Another had milk which stood on the streets at 97 on October 23, 1889, and the same day at dairy at 115. As 97 was a little high to prosecute, and as he had only a few cows, I took satisfaction out of him by publishing my report to the Board of Health giving the facts; since which time his milk stands in the neighborhood of 110. While milk that stands at 100, passes, the milk-man whose milk stands at that on the street, and at from 110 to 118 at his diary the same day, cannot long stand the publication of these facts.

A standard temperature of 60° Fahrenheit is agreed upon for lactometric testing of milk, and this is to be understood in the tests given.

Cows giving milk rich in cream, generally give milk rich in solids not fat. Consequently, Jersy milk will, as a rule, pass the standard.

Having lived on a farm and ranch in Texas, and raised dairy cattle eight years, I had practical experience of great value. I

neglected to say that I inspect dairies as to cleanliness, arrangements, etc., and also the cows as to health and condition.

The average man is interested in the work of the Board of Health when he can see that it pays. I am in a position to prove that the people of this city were paying at least $6,000 a year for added water in their milk when I commenced inspecting, in October last. This is saved to them now, and is double the amount considered necessary for sanitation next year by a very liberal council.

